# An exceptional case of bilateral gestational gigantomastia with multiple breast lumps

**DOI:** 10.11604/pamj.2015.20.309.6544

**Published:** 2015-03-31

**Authors:** Bangaly Traoré, Bakarou Kamate, Mamoudou Conde, Ahmed Monzomba Keita, Tidiane Kourouma, Ahmadou Dem

**Affiliations:** 1 Unit of Surgical Oncology, Donka University Hospital Centre, Conakry, Guinea; 2 Laboratory of Pathology, Hospital Point G, Bamako, Mali; 3 Laboratory of Pathology, Hospital Sino-Guinéen, Conakry, Guinea; 4 Cancer Institute, Dakar, Senegal

**Keywords:** Gestational gigantomastia, multiple breast lumps, lumpectomy

## Abstract

Bilateral gigantomastia is a rare condition, often associated with pregnancy that is characterized by a diffuse enlargement of both breasts. Here we present a case of a late 20s woman in her seven months pregnancy with a bilateral gestational gigantomastia associated with multiple breast lumps. Histological analysis revealed a fibroadenoma. Her prolactin level after caesarean delivery was found particularly high. A significant decrease in breast size was achieved with bromocriptine treatment in conjunction with a bilateral lumpectomy. This case report highlights the diversity of gigantomastia and emphasizes the importance of a tailored, multidisciplinary approach to the diagnosis and treatment of this condition.

## Introduction

Gigantomastia is an excessive, diffuse increase in breast volume. It is a very rare mammary gland condition with a prevalence of 1 case per 100 000 pregnancies [1]. The hormonal aetiology of gigantomastia remains controversial. The clinical signs of the condition, including inflammation and breast pain, can be similar to those of inflammatory breast cancer [2]. To the best of our knowledge, a case of gestational gigantomastia occurring in association with breast lumps has never been described. This case report discusses the diagnostic and therapeutic approaches taken with an unusual case of gestational gigantomastia with breast lumps, and emphasizes the importance of a tailored, multidisciplinary approach to the management of this diverse condition.

## Patient and observation

A seven months pregnant woman in her late 20s from a small town in northern Guinea was hospitalized at a clinic in Conakry for bilateral gigantomastia associated with multiple breast lumps. The patient reported that the condition had been present for about 3 months, i.e. approximately at four months of gestational amenorrhea. The patient was in her third pregnancy and she never experienced before any size abnormality of the breasts. In addition to the gigantomastia, the patient complained of breast itch and a severe pain in the breasts and back. At the physical examination, the patient was found pale with a pregnancy of 31 weeks and had a bilateral gigantomastia, predominantly in the right breast. The axillary tail of breast tissue was curved. Both breasts had hyperpigmentation and were sensitive to palpation. The nipple skin was thickened. There was a firm straight axillary lymphadenopathy (hard, with certain mobility of lymph nodes) but no supraclavicular node. Breast ultrasonography showed multiple hypoechogenic masses with posterior enhancement in the axillary tail of both breasts and in the inner lower quadrant of the right breast. There was an evolutive singleton pregnancy of 31 weeks. Fine-needle aspiration and core biopsy analysis of the breast lumps revealed atypical epithelial hyperplasia. The patient was also suffering of severe anemia with an haemoglobin concentration of 7.5 g/dL. HIV serology was negative. Blood tests performed after delivery showed the following hormone levels: prolactin 111.1 ng/mL, oestradiol 15.8 pg/mL, progesterone 2.4 mg/mL, luteinizing hormone 0.13 mIU/mL, and follicle-stimulating hormone 0.10 mIU/mL. These values are consistent with hyperprolactinemia and hyperoestradiolémia.

**Figure 1:**
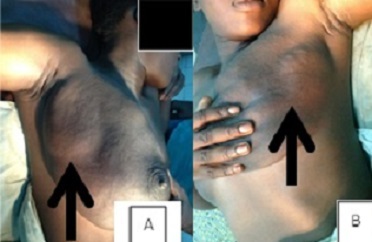
(A) appearance of bilateral gigantomastia associated with lumps (arrows) in the right; (B) left breasts

**Figure 2:**
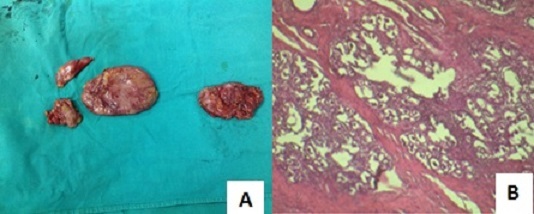
(A) surgical specimens from the lumps in the boths breasts; (B) histology of the resected lumpectomy specimen (H&E), magnified view × 4, showed fibroadenoma with galactophorous duct and fibrous tissue proliferation

**Figure 3:**
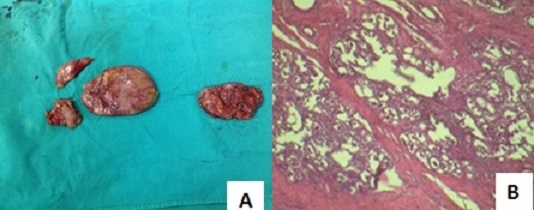
(A) appearance of the breasts at the time of the diagnosis; (B) after bromocriptine and surgical treatment

To manage the patient's breast and back pain, which would have been exacerbated by vaginal delivery, a prophylactic caesarean section was performed at 34 weeks gestation, after steroid administration to accelerate fetal lung maturation. A live male infant weighing 2.3 kg was delivered. Bromocriptine treatment was then initiated (at a dosage of half a 2.5-mg per tablet on day 1, one tablet on day 2, two tablets on day 3, and then increasing by one tablet per day to a maximum of 10 mg per day for 15 days). A significant reduction in breast volume was achieved with bromocriptine treatment (Figure 1), revealing easily palpable breast lumps. Bromocriptine treatment during fifteen days also resulted in a fall in prolactin levels to 19ng/mL. Side effects of bromocriptine were headache relieved by tramadol. Bilateral lumpectomy was performed two months after bromocriptine treatment. The excised lumps were well circumscribed and varied in size (Figure 2). The patient's postsurgical follow-up was marked by delayed healing due to a haematoma. Histological examination of the surgical specimens confirmed fibroadenoma (Figure 2). Both breasts returned to a normal size compared to the initial size at the time of diagnosis (Figure 3). After 6 months of breast lumpectomy, there was no relapse. In addition, breast and back pain were relieved after delivery.

## Discussion

The association of breast lumps with bilateral gestational gigantomastia is exceptional. Only one other case of gigantomastia with breast lumps has been reported, and that case occurred after kidney transplantation [3]. Rare cases of gigantomastia associated with breast tumours have been reported, but only in the context of unilateral gigantomastia [4–6]. This case report describes a case of bilateral gestational gigantomastia predominantly in the right breast and with lumps in both breasts. Asymmetry in breast size in bilateral gestational gigantomastia has also been reported by Ezem et al [7]. Cesarean was justified by the unbearable back and breast pain. It can also prevent in some case complications of the breast infection [1]. The primary treatment indicated was medical, involving the administration of bromocriptine [2]. The use of this drug was very efficient, as a significant decrease in the size of the breasts occurred during treatment. However, in some cases of gestational gigantomastia, breast size decreases spontaneously [7]. Mastectomy with breast reconstruction is indicated in the absence of gigantomastia resolution after delivery and also in the case of relapse or complications during pregnancy [8, 9]. In this case, in which favourable results were achieved with bromocriptine treatment, lumpectomy was indicated due to the persistence of nodules in both breasts. A limitation of this case report is the lack of information about the patient´s hormone levels before delivery. In addition, there was no histological examination of the breast tissue surrounding the lumps. The availability of these data in future cases could help clinicians make the decision to start treatment with bromocriptine before delivery, and could also facilitate the differential diagnosis of this condition from other breast diseases. Indeed, the presence of skin inflammatory signs, axillary lymphadenopathy, and atypia (found on fine-needle aspiration) may have suggested a case of inflammatory breast carcinoma, with a possibility of phyllodes tumours. However, the clinical outcome after delivery, i.e. the decrease in the size of both breasts with bromocriptine treatment, the clinical characteristics of the breast lumps, and particularly the histology of the lumps led to a confirmed diagnosis of fibroadenoma.

## Conclusion

This report describes the first known case of gestational gigantomastia associated with multiple fibroadenomas; however, the role of such fibroadenomas in the genesis or exacerbation of gigantomastia remains to be determined.
